# Supplementary material for: Nucleoporin downregulation modulates progenitor differentiation independent of nuclear pore numbers
Source: Commun Biol. 2023 Oct 18;6:1033. doi: 10.1038/s42003-023-05398-6 (PMC10584948; doi:10.1038/s42003-023-05398-6)

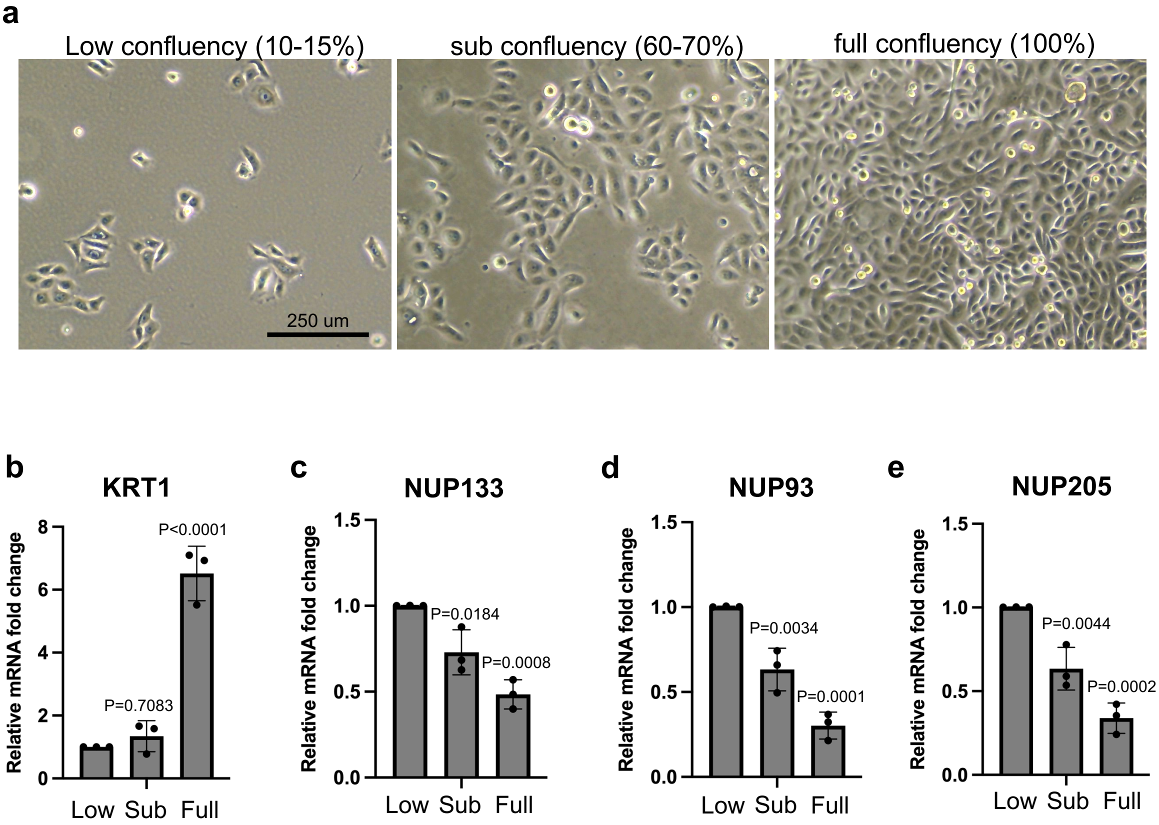


**Supplementary Fig. 1. NUP downregulation in confluence-induced early differentiation.** (**a**) Representative phase-contrast images of keratinocytes growing at low, sub, or full confluency. (**b-e**) qRT-PCR quantification of KRT1, NUP133, NUP93 or NUP205 relative mRNA expression in keratinocytes growing in low, sub, and full confluency (one-way ANOVA with post-hoc test, N = 3 biological replicates, data are represented as mean ± standard deviation).

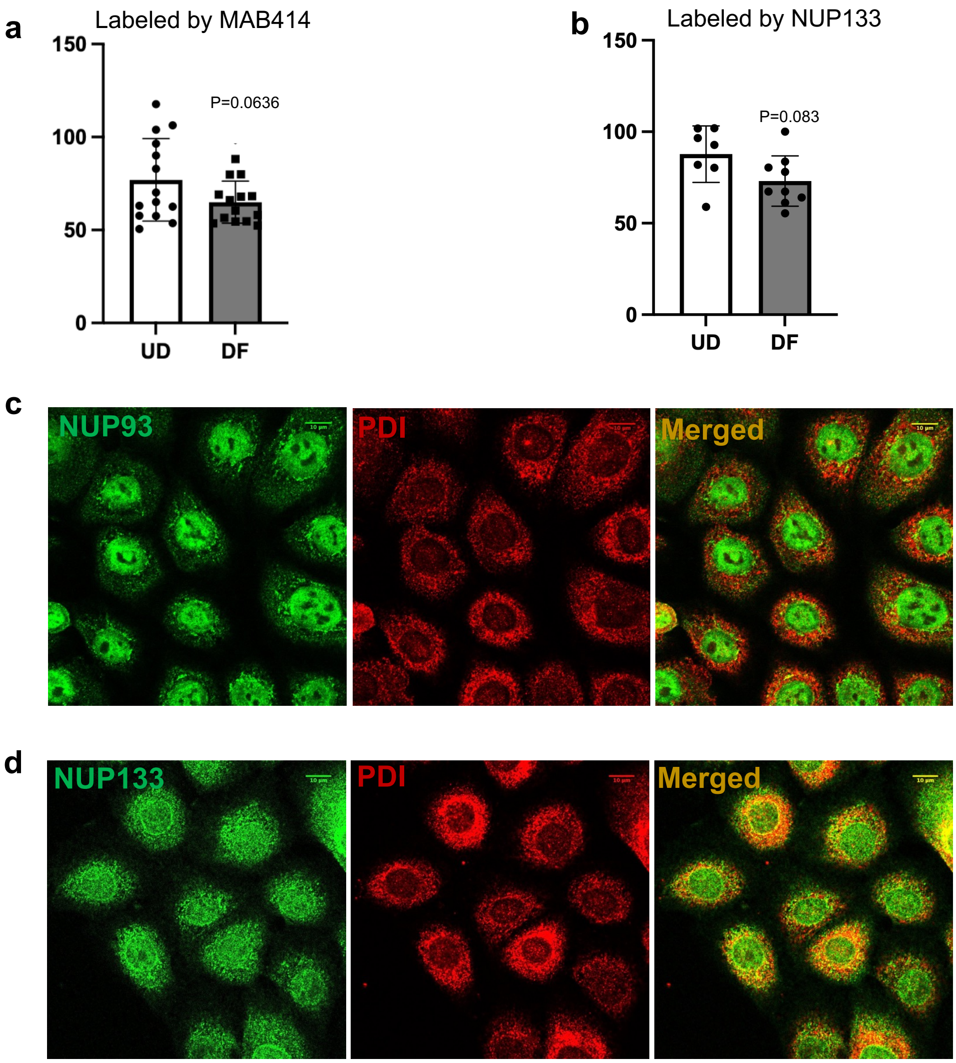


**Supplementary Fig. 2. Size comparison of extracted nuclei from keratinocytes in the progenitor state versus the differentiation state (day 4).**
(**a**) The maximum 2D area size of each extracted nucleus, from progenitor-state or differentiation-state keratinocytes, was recorded based on the fluorescence from MAB414 antibody labeling. The area size of each 2D image was quantified using image J, and the area sizes obtained in UD (n=14) vs DF (n=14) states were compared. p=0.0636, t-test. (**b**) Comparison of maximum 2D area sizes of extracted nuclei between UD (n=7) and DF (n=9), based on the fluorescence from NUP133 labeling. p=0.083, t-test. (**c, d**) Confocal images showing the subcellular localization of NUP133 (green) and NUP93 (green) with a cytoplasmic marker PDI (red). Scale bar=10uM.

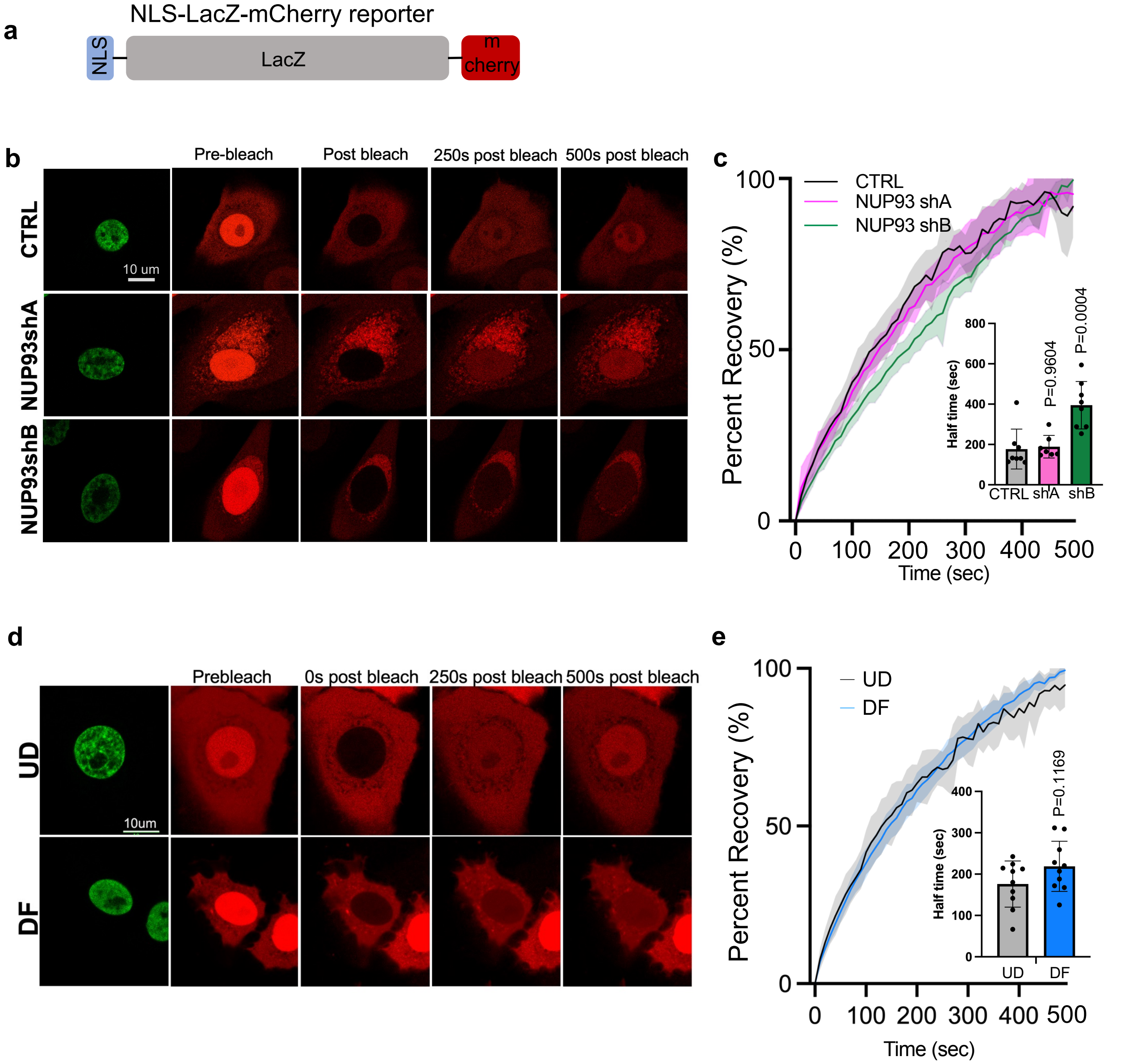


**Supplementary Fig. 3. No drastic changes in nuclear import with NUP93 reduction.**
(**a**) Illustration of the NLS-LacZ-mCherry reporter for assessing nuclear import using FRAP. (**b**) Representative images from the FRAP experiment comparing keratinocytes with NUP93 knockdown versus the control. (**c**) Fluorescence recovery trajectory of NLS-LacZ-mCherry in the nucleus after photobleaching, comparing keratinocytes with control shRNA (n=8) or NUP93 shRNA (n=7 for each shRNA). Lines and the shaded areas represent the average and standard errors. Comparisons of the Half time of recovery is included in the bar graph. (**d**) Representative images from the FRAP experiment comparing keratinocytes the progenitor state (UD) versus the differentiation state (DF). (**e**) Fluorescence recovery trajectory of NLS-LacZ-mCherry in the nucleus after photobleaching, comparing UD keratinocytes (n=10) and DF keratinocytes (n=10). Lines and the shaded areas represent the average and standard errors. Comparisons of the Half time of recovery is included in the bar graph.

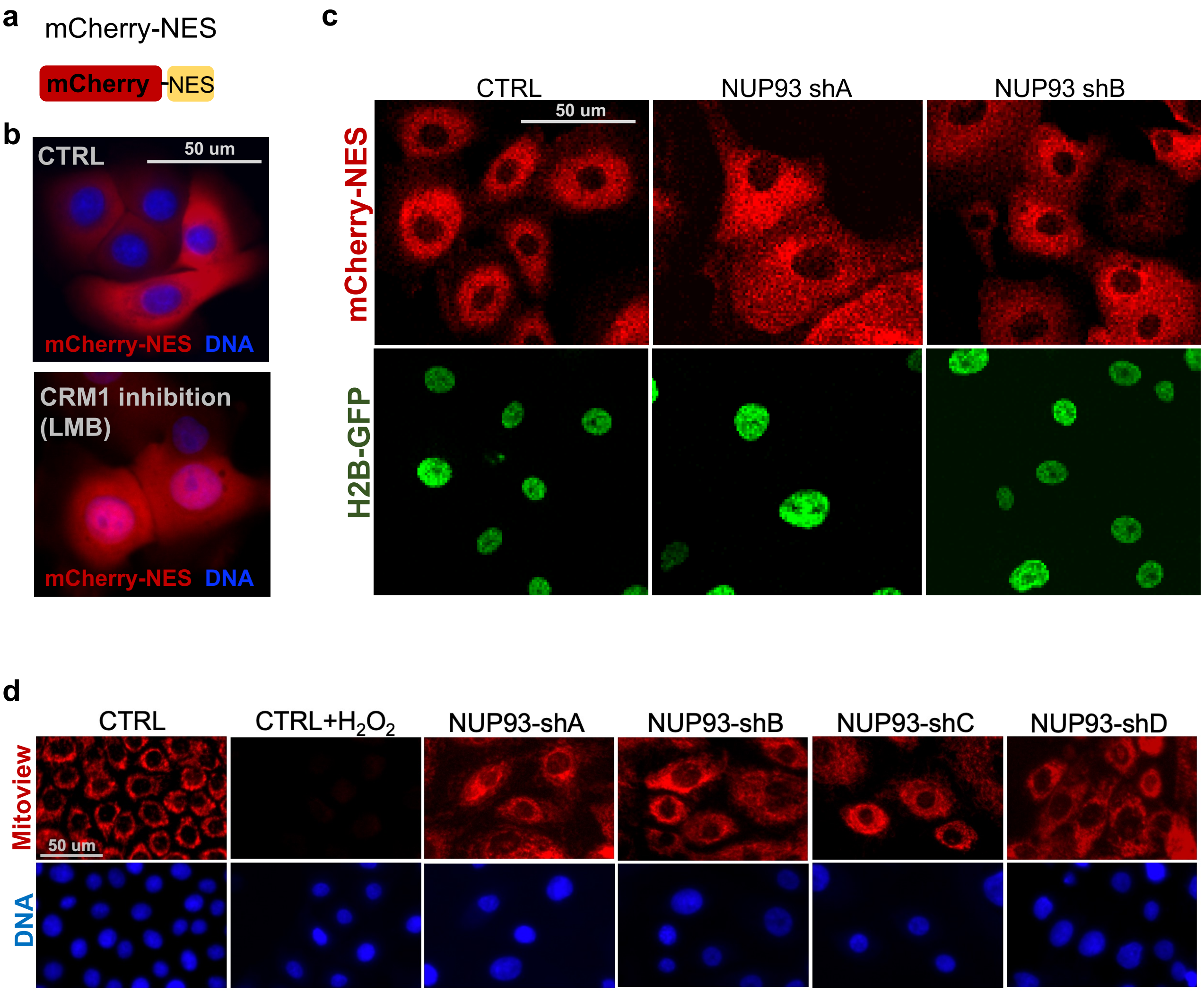


**Supplementary Fig. 4. NUP93 knockdown does not drastically affect nuclear export or apoptosis.**
(**a**) Schematic illustration of the mCherry-NES reporter used for assessing nuclear export. (**b**) Representative images of keratinocytes expressing the NES-mCherry reporter, with or without inhibiting the protein-export-regulator CRM1 using LMB. CRM1 inhibition led to nuclear translocation of this NES-mCherry reporter. (**c**) Representative images of keratinocytes expressing the NES-mCherry reporter, with or without NUP93 knockdown. This NES-mCherry reporter remains predominantly cytoplasmic with NUP93 knockdown. (**d**) Representative images of apoptosis assay using Mitoview. $H_2O_2$-treated control cells are used as positive control for apoptosis induction. No drastic differences were detected between control versus NUP93 knockdown.

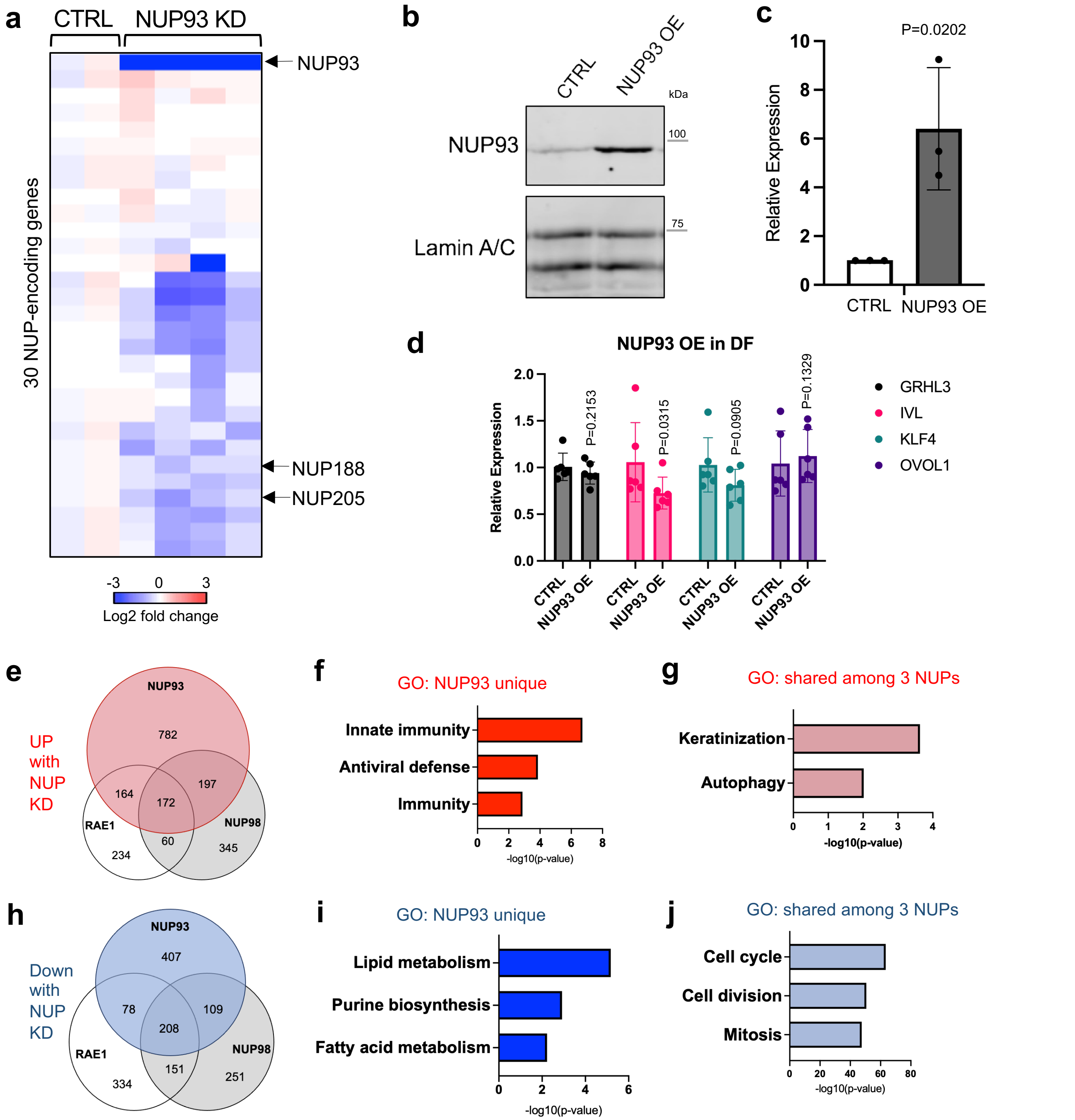


**Supplementary Fig. 5. NUP93 KD does not alter NPC formation, NUP expression, or induce apoptosis.**
(**a**) Heatmap showing the relative expression of of 30 NUP-encoding genes with control or NUP93 knockdown. (**b**) Western showing NUP93 overexpression (OE) in keratinocytes. (**c**) Quantification of NUP93 in overexpression western blot relative to the lamin loading control represented as the average +/- DF, N=3, t-test. (**d**) qRT-PCR quantification of representative differentiation genes comparing control differentiated keratinocytes (n=4) versus differentiated NUP93 OE (n=4) cells (t test, data are represented as mean ± standard deviation). (**e-j**) Comparisons of upregulated or downregulated genes with knockdown of NUP93, NUP98 or RAE1. The number of overlapping genes are indicated in the Venn diagram, and the GO terms of the shared or NUP93 unique genes are shown in the bar graphs.

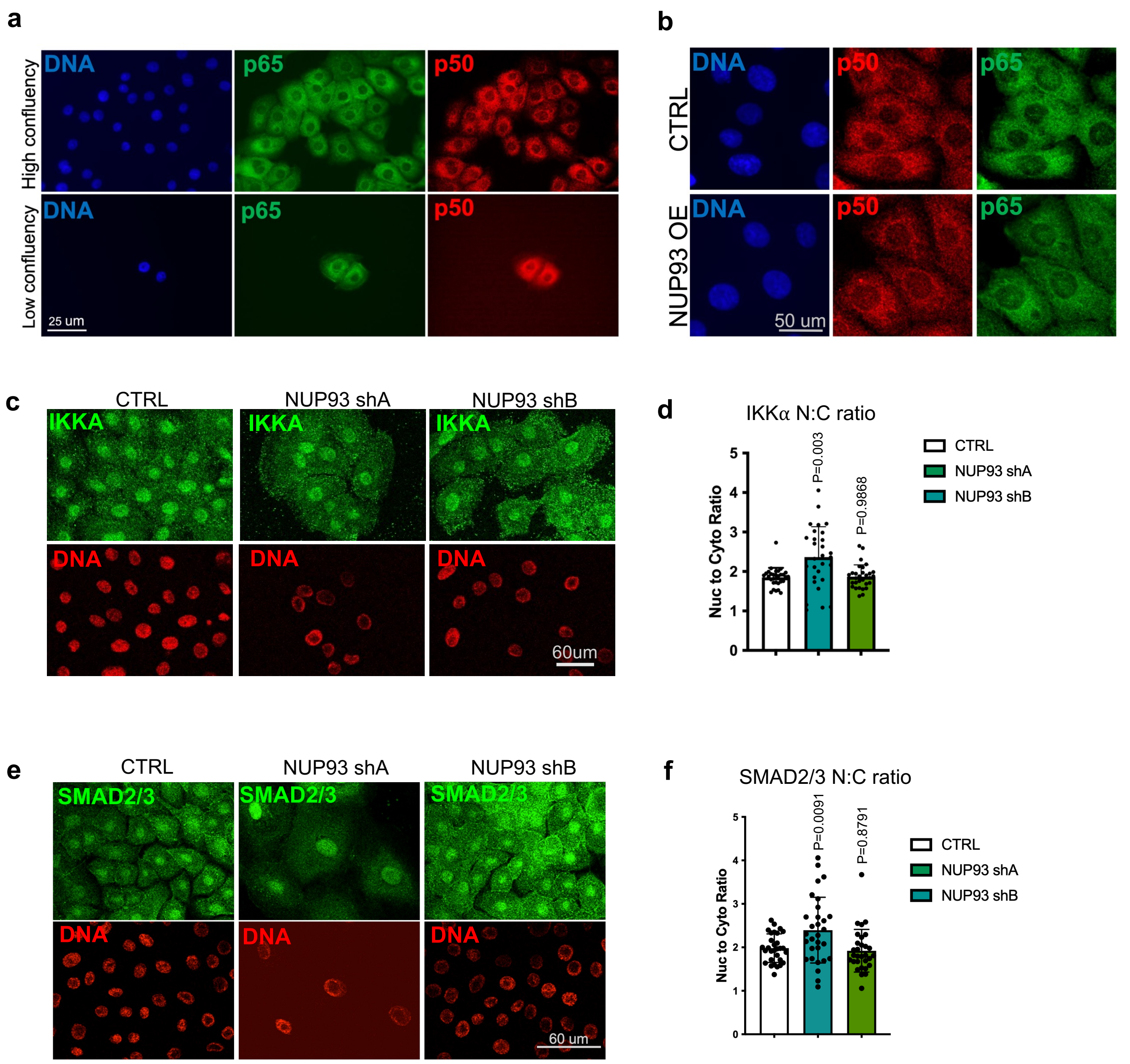


**Supplementary Fig. 6. Subcellular localization of p65/p50 is not drastically changed with increased confluency or with NUP93 overexpression.**
(**a**) Representative images of p65 and p50 in keratinocytes with high versus low cell confluency. (**b**) Representative images of p65 and p50 in keratinocytes with NUP93 overexpression versus control. (**c**) Representative images of IKKA immunostaining in keratinocytes, comparing NUP93 knockdown versus control knockdown. (**d**) Quantification of the relative ratio of fluorescence in the nucleus versus cytoplasm in each cell (N:C ratio). (CTRL vs NUP93-shA: p=0.003, t-test; CTRL vs NUP93-shB: p=0.98, t-test; n=30/condition). (**e**) Representative cell images of SMAD2/3 immunostaining in keratinocytes with NUP93 knockdown or control knockdown. (**f**) Quantification of the relative N:C ratio of SMAD2/3 in keratinocytes with NUP93 knockdown or non-targeting control knockdown. (CTRL vs NUP93-shA: p=0.009, t-test; CTRL vs NUP93-shB: p=0.879, t-test; n=30/condition).

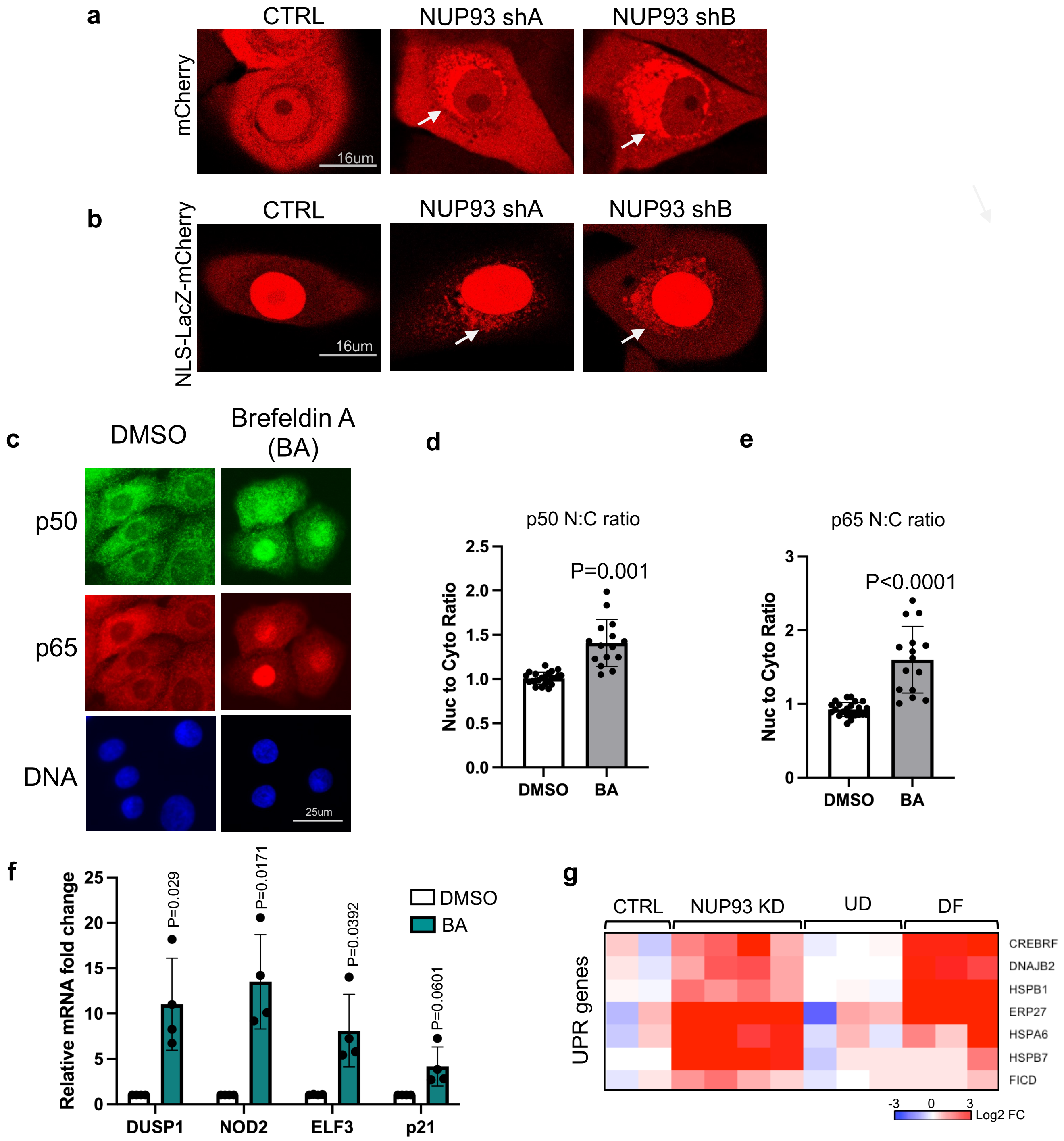


**Supplementary Fig. 7. NUP93 reduction involves stress response in keratinocytes.**
(**a, b**) Representative images showing keratinocytes expressing mCherry or NLS-lacZ-mCherry, with NUP93 knockdown or control knockdown. Arrowheads indicate protein aggregation outside the nucleus. (**c**) Representative images of p65 or p50 immunofluorescence staining, in keratinocytes treated with Brefeldin A (BA) or DMSO. (**d-e**) Quantification of the nuclear versus cytoplasmic localization of p50 or p65, in keratinocytes with BA treatment versus DMSO control. Data represented as the average +/- SD, N>/=15, t-test. (**f**) RT-qPCR quantification of representative differentiation genes with BA treatment versus control. Data represented as the average +/- SD, N=4, t-test. (**g**) Heatmap showing the relative expression of unfolded protein response genes in keratinocytes with NUP93 knockdown or in differentiation.

**Supplementary Fig. 8: uncropped westerns**

**Fig.1 g**

NUP205

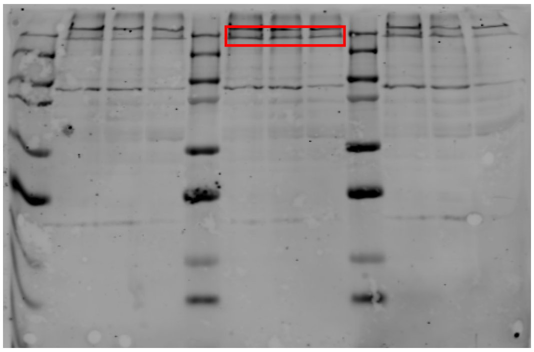

Lamin

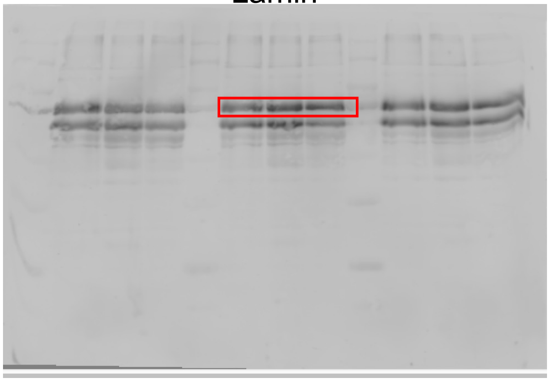

**Fig.1 h**

NUP93

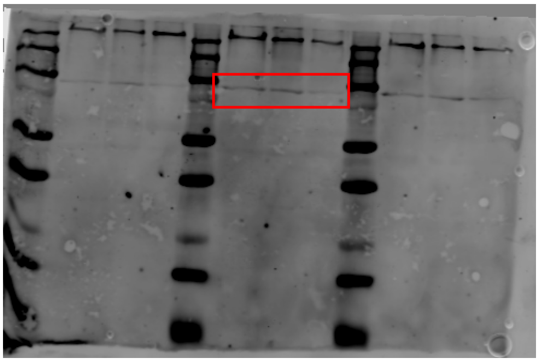

Lamin

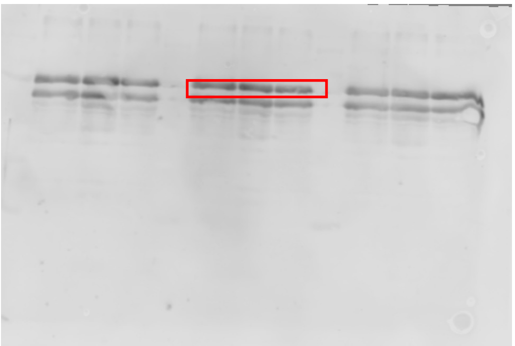

**Fig.1 i**

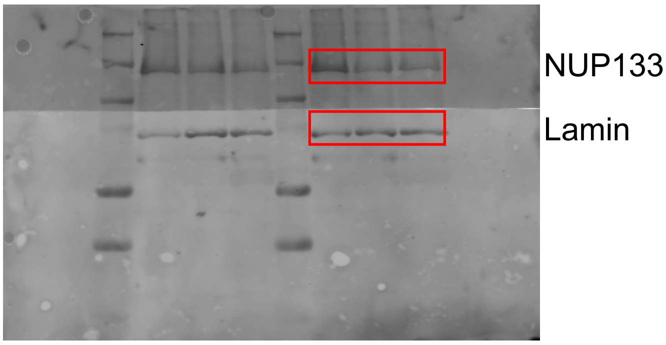

NUP133

Lamin

Supplementary Fig. 8: uncropped westerns (continued)

**Fig. 3j**

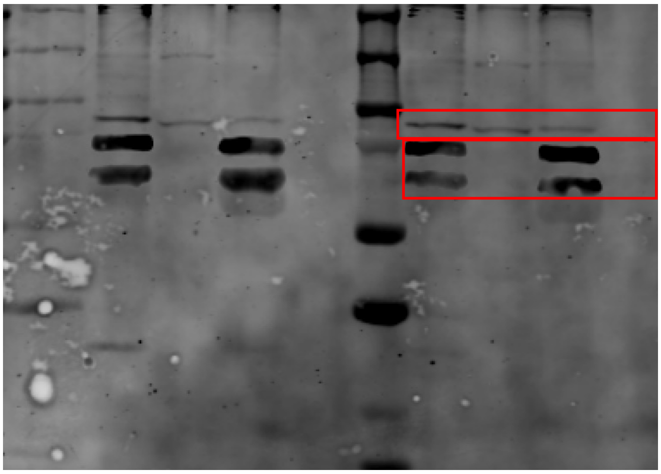


**Fig. 3m**

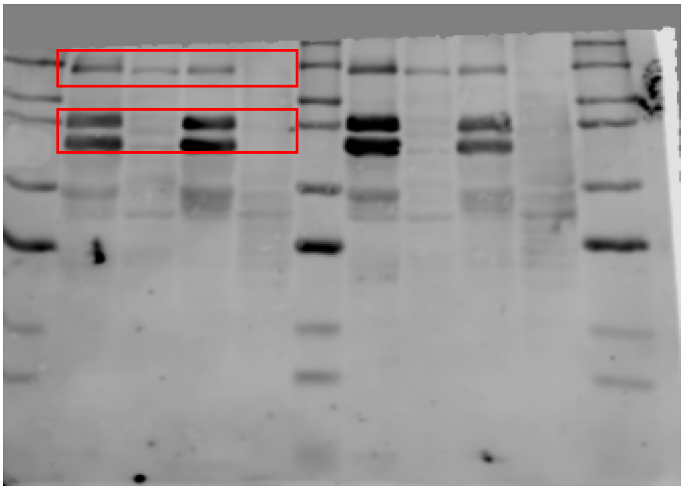


**Fig. 4b**

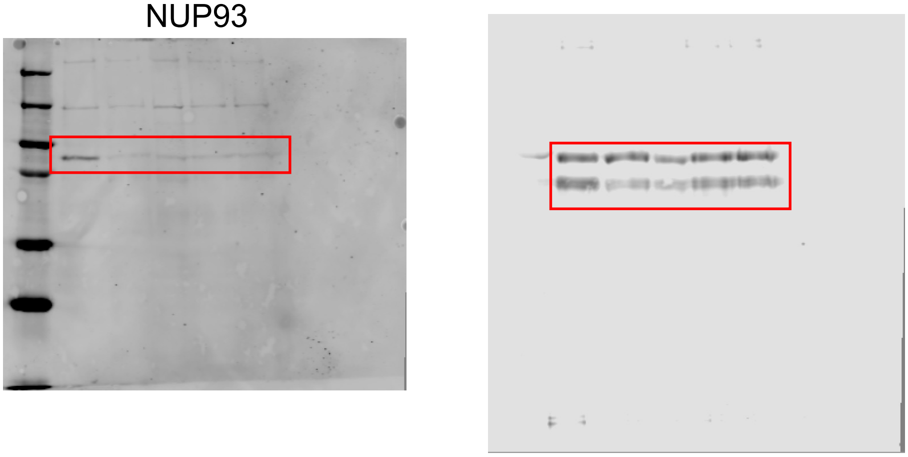

Supplementary Fig. 8: uncropped westerns (continued)

**Supplementary Fig. 5b**

NUP93

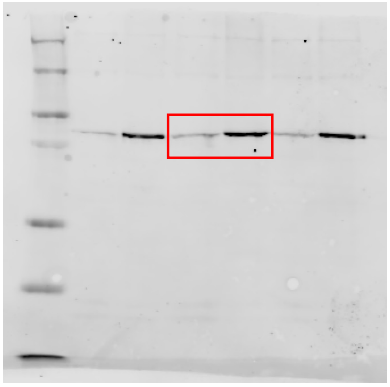

Lamin

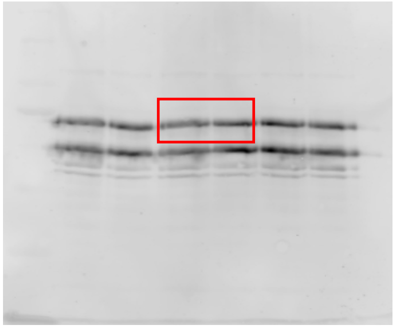

Supplement: Supplementary file 2 — supplementary figures [file 42003_2023_5398_MOESM2_ESM.pdf]
