# Supplementary material for: Nucleoporin downregulation modulates progenitor differentiation independent of nuclear pore numbers
Source: Commun Biol. 2023 Oct 18;6:1033. doi: 10.1038/s42003-023-05398-6 (PMC10584948; doi:10.1038/s42003-023-05398-6)
Supplement: Supplementary file 3 — Description of Supplementary Materials [file 42003_2023_5398_MOESM3_ESM.docx]

**Description of Additional Supplementary Files**

**File name:** Supplementary Data 1

**Description:** Differentially Expressed (DE) genes with NUP93 knockdown

**File name:** Supplementary Data 2

**Description:** List of Oligos used in this study

**File name:** Supplementary Data 3

**Description:** Source data
