# Supplementary material for: Nucleoporin downregulation modulates progenitor differentiation independent of nuclear pore numbers
Source: Commun Biol. 2023 Oct 18;6:1033. doi: 10.1038/s42003-023-05398-6 (PMC10584948; doi:10.1038/s42003-023-05398-6)
Supplement: Supplementary file 7 — Reporting Summary [file 42003_2023_5398_MOESM7_ESM.pdf]

nature portfolio

Corresponding author(s): Xiaomin Bao

Last updated by author(s): Sep 20, 2023

Reporting Summary

Nature Portfolio wishes to improve the reproducibility of the work that we publish. This form provides structure for consistency and transparency in reporting. For further information on Nature Portfolio policies, see our Editorial Policies and the Editorial Policy Checklist.

Statistics

For all statistical analyses, confirm that the following items are present in the figure legend, table legend, main text, or Methods section.

n/a	Confirmed	
☐	☒	The exact sample size (*n*) for each experimental group/condition, given as a discrete number and unit of measurement
☐	☒	A statement on whether measurements were taken from distinct samples or whether the same sample was measured repeatedly
☐	☒	The statistical test(s) used AND whether they are one- or two-sided<br>*Only common tests should be described solely by name; describe more complex techniques in the Methods section.*
☐	☒	A description of all covariates tested
☐	☒	A description of any assumptions or corrections, such as tests of normality and adjustment for multiple comparisons
☐	☒	A full description of the statistical parameters including central tendency (e.g. means) or other basic estimates (e.g. regression coefficient) AND variation (e.g. standard deviation) or associated estimates of uncertainty (e.g. confidence intervals)
☐	☒	For null hypothesis testing, the test statistic (e.g. *F*, *t*, *r*) with confidence intervals, effect sizes, degrees of freedom and *P* value noted<br>*Give P values as exact values whenever suitable.*
☒	☐	For Bayesian analysis, information on the choice of priors and Markov chain Monte Carlo settings
☒	☐	For hierarchical and complex designs, identification of the appropriate level for tests and full reporting of outcomes
☒	☐	Estimates of effect sizes (e.g. Cohen's *d*, Pearson's *r*), indicating how they were calculated

Our web collection on statistics for biologists contains articles on many of the points above.

Software and code

Policy information about availability of computer code

Data collection	The qRT-PCR data were acquired using QuantStudio Design & Analysis Software 1.3.1 (Thermo Fisher)<br>Western blots images were collected using Image studio software version 5.2 (LI-COR)<br>Immunofluorescence images were collected using the EVOS FL Auto 2 Imaging system software (Thermo Fisher)
Data analysis	This paper does not report original code.<br>Quantification of western blots was done using Image studio software version 5.2 (LI-COR).<br>Immunofluorescence images were analyzed using ImageJ version 2.1.0<br>Statistical analysis for qPCR, Western, and Immunofluorescence was done using GraphPad Prism version 9.<br>For RNA-seq the software is described in the Methods section and R Studio version 3.5.2 was used for analysis. Other softwares used include DAVID Gene Ontology analyses.

For manuscripts utilizing custom algorithms or software that are central to the research but not yet described in published literature, software must be made available to editors and reviewers. We strongly encourage code deposition in a community repository (e.g. GitHub). See the Nature Portfolio guidelines for submitting code & software for further information.

Data

Policy information about availability of data

All manuscripts must include a data availability statement. This statement should provide the following information, where applicable:

- Accession codes, unique identifiers, or web links for publicly available datasets
- A description of any restrictions on data availability
- For clinical datasets or third party data, please ensure that the statement adheres to our policy

The RNA-seq data have been deposited to GEO, with the accession number #GSE209655 (including both GSM7507285-GSM7507290). The reviewer token is kfofieksnngbfcd.

Human research participants

Policy information about studies involving human research participants and Sex and Gender in Research.

Reporting on sex and gender	Keratinocytes were isolated from surgically discard skin specimen from de-identified donors. Keratinocytes isolated from 6 donors were pooled for all the experiments performed in this study. Therefore, this study is not focused on the characterization of cells from individual donors.
Population characteristics	All the experiments were performed using pooled primary keratinocytes pooled from 6 de-identified donors, at early passages. The purity of each batch of primary keratinocytes were confirmed based on cell morphology using microscopy.
Recruitment	Not applicable
Ethics oversight	This research was reviewed by Northwestern University Institute Review Board (IRB) and assigned a determination of NOT HUMAN RESEARCH.

Note that full information on the approval of the study protocol must also be provided in the manuscript.

Field-specific reporting

Please select the one below that is the best fit for your research. If you are not sure, read the appropriate sections before making your selection.

☒ Life sciences ☐ Behavioural & social sciences ☐ Ecological, evolutionary & environmental sciences

For a reference copy of the document with all sections, see nature.com/documents/nr-reporting-summary-flat.pdf

Life sciences study design

All studies must disclose on these points even when the disclosure is negative.

Sample size	Primary human keratinocytes were pooled from at least six donors, as is standard for primary keratinocyte culture (Bao et al, 2017). Gene knockdown experiments were done using two non- targeting controls and two or three independent shRNA targeting the gene of interest as was done in Bao et al 2017. RT-qPCR experiments were all done with at least three biological replicates and each biological replicate was done in technical triplicates. RNA-seq data was also done in biological duplicate or triplicates. For tissue regeneration assay, at least two organotypic cultures were preprepared for each sample (Li et al, 2021). All immunofluorescence data were done in biological triplicates and a minimum of three images were used for quantification between replicates. All replicates showed the same trend; one representative image was shown.
Data exclusions	No data were excluded from analysis.
Replication	All experiments were performed in either technical or biological replicates. All replicates generated were used for this paper.
Randomization	Six independent (de identified) donors were used for pooling primary human keratinocytes for all experiments.
Blinding	Blinding was not required for this study.

Reporting for specific materials, systems and methods

We require information from authors about some types of materials, experimental systems and methods used in many studies. Here, indicate whether each material, system or method listed is relevant to your study. If you are not sure if a list item applies to your research, read the appropriate section before selecting a response.

Materials & experimental systems

n/a	Involved in the study
☐	☒ Antibodies
☐	☒ Eukaryotic cell lines
☒	☐ Palaeontology and archaeology
☒	☐ Animals and other organisms
☒	☐ Clinical data
☒	☐ Dual use research of concern

Methods

n/a	Involved in the study
☒	☐ ChIP-seq
☒	☐ Flow cytometry
☒	☐ MRI-based neuroimaging

Antibodies

Antibodies used

Antibodies used for western in this study include NUP93 E-8 (Santa Cruz Biotechnology, sc-374399), Lamin A/C (Santa Cruz Biotechnology, sc-376248), NUP205 (H1) (Santa Cruz sc-377047), NUP133 (Santa Cruz sc-376699), p65 (Cell Signaling, #8242), p50(E-10) (Santa Cruz, sc-8414), mab414 (BioLegend, MMS-120p)

Validation

NUP93 E-8 (Santa Cruz Biotechnology, sc-374399) was validated for WB, IP, IF, IHC(P) and ELISA by manufacturer. This antibody has been cited 9 times. The information can be found at: https://www.scbt.com/p/nup93-antibody-e-8.

Lamin A/C (Santa Cruz Biotechnology, sc-376248) was validated for WB, IP, IF, IHC(P) and ELISA by manufacturer. This antibody was been cited 206 times. The information can be found at: https://www.scbt.com/p/lamin-a-c-antibody-e-1.

NUP205 (H1) (Santa Cruz sc-377047) was validated for WB, IP, IF and ELISA by manufacturer. This antibody has been cited 2 times. The information can be found at: https://www.scbt.com/p/nup205-antibody-h-1.

NUP133 (Santa Cruz sc-376699) was validated for WB, IP, IF and ELISA by manufacturer. This antibody has been cited 3 times. The information can be found at: https://www.scbt.com/p/nup133-antibody-e-4.

p65 (Cell Signaling, #8242) was validated for WB, IP, IF and ChIP by manufacturer. This antibody has been cited 4,682 times. The information can be found at: https://www.cellsignal.com/products/primary-antibodies/nf-kb-p65-d14e12-xp-rabbit-mab/8242.

p50(E-10) (Santa Cruz, sc-8414) was validated for WB, IP, IF, IHC(P), FCM and ELISA by manufacturer. This antibody has been cited 347 times. The information can be found at: https://www.scbt.com/p/nfkappab-p50-antibody-e-10.

mab414 (BioLegend, MMS-120p) was validated for ICC, WB, IHC, and IP by manufacturer. This antibody has been cited 45 times. The information can be found at: https://www.biolegend.com/en-us/products/purified-anti-nuclear-pore-complex-proteins-antibody-11498.

PDI (Cell signaling #2446) was validated for WB, IHC, and IF by the manufacturer. This antibody is associated with 60 citations. The information can be found at: https://www.cellsignal.com/products/primary-antibodies/pdi-antibody/2446?_requestid=2770167

Eukaryotic cell lines

Policy information about cell lines and Sex and Gender in Research

Cell line source(s)	Primary human keratinocytes were used for all experiments. Cell lines were not used for experimental data or conclusions in this study. 293T or phoenix cells were used for lenti/retro virus production to infect primary human keratinocytes with trans gene. Clonogenicity assays were done using 3T3 cells as feeder cells. These cell lines were generous gifts from the Khavari Lab at Stanford University. The cell lines are also available commercially from ATCC (CRL-3216, CRL-1658, CRL-3213).
Authentication	Cell lines were authenticated by morphology.
Mycoplasma contamination	The "MycoFLuor Mycoplasma Detection Kit" (cat#M7006) was used to test all cell lines and primary human keratinocytes. The results for all tested cell lines was negative for mycoplasma.
Commonly misidentified lines (See ICLAC register)	No commonly misidentified cell lines were used in this study.
